# Immunohistochemical Assessment of Expression of Centromere Protein—A (*CENPA*) in Human Invasive Breast Cancer

**DOI:** 10.3390/cancers3044212

**Published:** 2011-12-06

**Authors:** Ashish B. Rajput, Nianping Hu, Sonal Varma, Chien-Hung Chen, Keyue Ding, Paul C. Park, Judy-Anne W. Chapman, Sandip K. SenGupta, Yolanda Madarnas, Bruce E. Elliott, Harriet E. Feilotter

**Affiliations:** 1 Department of Pathology and Molecular Medicine, Queen's University, Kingston, ON K7L 3N6, Canada; E-Mails: rajputa@queensu.ca (A.B.R.); 6sd37@queensu.ca (S.V.); chenj@kgh.kari.net (C.H.C.); 8pcp1@queensu.ca (P.C.P.); sengupts@kgh.kari.net (S.K.S.); elliottb@queensu.ca (B.E.E.); 2 Cancer Research institute, Queen's University, Kingston, ON K7L 3N6, Canada; E-Mails: nphu@queensu.ca (N.H.); yolanda.madarnas@krcc.on.ca (Y.M.); 3 NCIC Clinical Trials Group, Queen's University, Kingston, ON K7L 3N6, Canada; E-Mails: kding@ctg.queensu.ca (K.D.); jchapman@ctg.queensu.ca (J.-A.W.C.); 4 Department of Oncology, Cancer Center of Southeastern Ontario, Kingston, ON K7L 2V7, Canada

**Keywords:** *CENPA*, breast cancer, immunohistochemistry

## Abstract

Abnormal cell division leading to the gain or loss of entire chromosomes and consequent genetic instability is a hallmark of cancer. Centromere protein –A (*CENPA*) is a centromere-specific histone-H3-like variant gene involved in regulating chromosome segregation during cell division. *CENPA* is one of the genes included in some of the commercially available RNA based prognostic assays for breast cancer (BCa)—the 70 gene signature MammaPrint^®^ and the five gene Molecular Grade Index (MGI^SM^). Our aim was to assess the immunohistochemical (IHC) expression of CENPA in normal and malignant breast tissue. Clinically annotated triplicate core tissue microarrays of 63 invasive BCa and 20 normal breast samples were stained with a monoclonal antibody against CENPA and scored for percentage of visibly stained nuclei. Survival analyses with Kaplan–Meier (KM) estimate and Cox proportional hazards regression models were applied to assess the associations between CENPA expression and disease free survival (DFS). Average percentage of nuclei visibly stained with CENPA antibody was significantly higher (p = 0.02) in BCa than normal tissue. The 3-year DFS in tumors over-expressing CENPA (>50% stained nuclei) was 79% compared to 85% in low expression tumors (<50% stained nuclei). On multivariate analysis, IHC expression of CENPA showed weak association with DFS (HR > 60.07; p = 0.06) within our small cohort. To the best of our knowledge, this is the first published report evaluating the implications of increased IHC expression of CENPA in paraffin embedded breast tissue samples. Our finding that increased CENPA expression may be associated with shorter DFS in BCa supports its exploration as a potential prognostic biomarker.

## 1. Introduction

Genomic stability depends upon equal partitioning of replicated chromosomes to daughter cells during cell division. An error in chromosome segregation leading to chromosome copy number changes is known as ‘aneuploidy’. The fact that about 90% of solid tumors are aneuploid [1] underscores the importance of accurately functioning mitotic cell cycle machinery.

During cell division, newly formed chromosomes are equally distributed between daughter cells. Errors during mitosis in somatic cells cause aneuploidy, which consequently can lead to carcinogenesis by altering the balance of oncogenes and tumor suppressors [2]. The kinetochore is a large complex of proteins and associated centromeric DNA that attaches the chromosomes to the spindle for proper movement through the replication process [3]. Centromere protein –A (CENPA) is a fundamental protein unit of centromeres. It is a centromere-specific histone-H3-like variant that distinguishes centromeric from other chromatin [4,5].

Published literature supports the view that carcinogenesis occurs when kinetochores become functionally unstable leading to abnormal segregation of chromosomes and consequent genetic instability [6,7]. It has been reported that CENPA over-expression could potentially lead to spreading of centromere heterochromatin along chromosome arms causing defects in microtubules-kinetochores anchoring and eventually causing genomic instability [8].

*CENPA* is one of the genes whose expression is measured in commercially available, mRNA based prognostic assays such as the 70 gene MammaPrint^®^ [9] and the 5 gene Molecular Grade Index (MGI^SM^) [10]. MammaPrint^®^ is a prognostic gene expression signature profile developed in 2001 at the Netherlands Cancer Institute (NKI) to help clinicians assess for the risk of recurrence and formulate a management plan to treat a growing population of patients with early stage breast cancer (BCa) [9]. By expression analysis, 231 genes were identified as correlated with BCa recurrence and the top ranked 70 genes were selected for the profile that has become the diagnostic MammaPrint^®^ test. The test identifies patients at either “low” or “high” risk of recurrence who would benefit from the addition of adjuvant chemotherapy, and those who could be managed with endocrine treatment alone [11].

*CENPA* is also one of the five cell-cycle genes (*BUB1B, CENPA, NEK2, RACGAP1*, and *RRM2*) whose expression is used to calculate the Molecular Grade Index (MGI^SM^). The MGI^SM^ was developed at Massachusetts General Hospital and validated as a five-gene reverse transcriptase polymerase chain reaction (RT-PCR) assay for analyzing formalin fixed paraffin embedded (FFPE) clinical samples. The MGI is a prognostic assay that classifies grade 2 tumors to be either grade 1–like or grade 3–like, removing ambiguity of pathologic tumor grading and reducing inter-observer variability. It identifies low-risk women who may be spared from toxic chemotherapy and a subgroup at high risk for whom more intense chemotherapy regimens or new therapeutic agents should be considered [10].

From a practical point of view, it is important to note that these tests determine the mRNA level from tumors, which can be problematic in the clinical setting because most clinical service laboratories are not experienced in the extraction and analysis of this highly labile macromolecule. Developing an immunohistochemical (IHC) assay for examining protein expression in the biomarkers of interest would make it feasible for most diagnostic labs to carry out the tests in a standardized manner. As an initial step, we undertook this study to assess the IHC expression of CENPA in FFPE BCa tissues, optimize cut-points to predict BCa specific survival and assess its correlation with standard clinico-pathological parameters.

## 2. Results and Discussion

### 2.1. Clinico-Pathologic Characteristics of the Study Population

The patients with primary BCa tumors in this tissue microarray (TMA) were all managed at the Kingston Regional Cancer Centre from 2005 to 2007, after the introduction of routine HER 2 testing. The clinical database included traditional prognostic factors [age, tumor size, lymph node status, histological type, grade, lymphovascular invasion, estrogen receptor (ER), progesterone receptor (PR) and human epidermal growth factor receptor-2 (HER 2) status], treatment information [surgery, adjuvant systemic therapy, adjuvant radiotherapy (RT)] as well as outcome information [date and sites of relapse, treatment at relapse, date and cause of death]. The patients were sequentially identified from the database, subject to meeting the pre-defined entry criteria. Patients included in the study were premenopausal (less than 49 years of age at diagnosis), had primary infiltrating ductal or lobular carcinomas and were stage T1-3a, N0-1, M0. Patients were excluded if they had any previous history of cancer, bilateral breast disease or neoadjuvant chemotherapy. Mean age of this patient cohort was 43.5 years, (range 29–49). The majority of the patients (60%) had N-0 disease and received adjuvant chemotherapy (74%). This small cohort contains each of the current molecularly defined BCa subtypes, and segregates as 16% basal-like/triple negative (ER-/PR-/HER 2-), 61% luminal A (ER+/PR+), 20% luminal B (ER+/PR+/HER 2+), and 3% HER 2 positive cases. It is important to note that the molecular subtypes in our cohort differ from the expected proportions reported in larger population based cohorts [12]. The patient characteristics are summarized in Table 1.

### 2.2. CENPA Expression in Breast Samples

**Figure 1.**
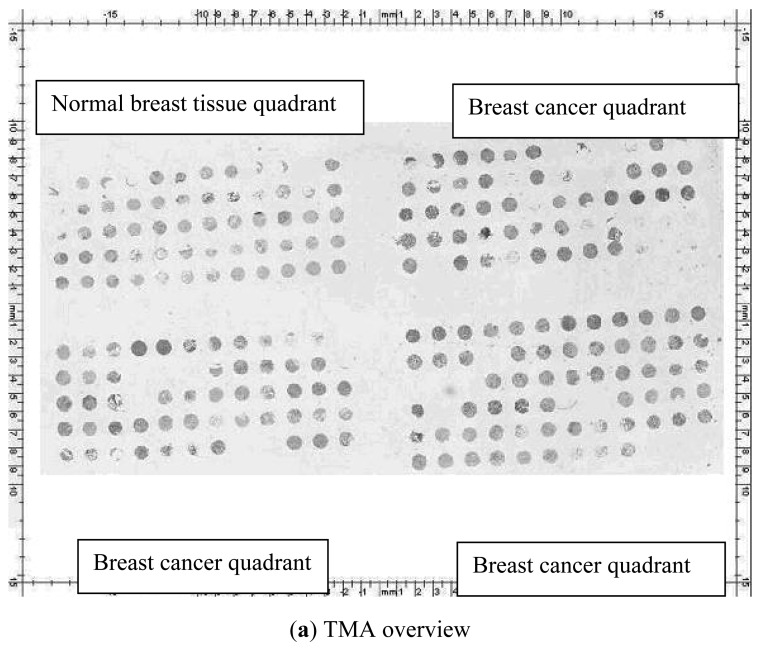

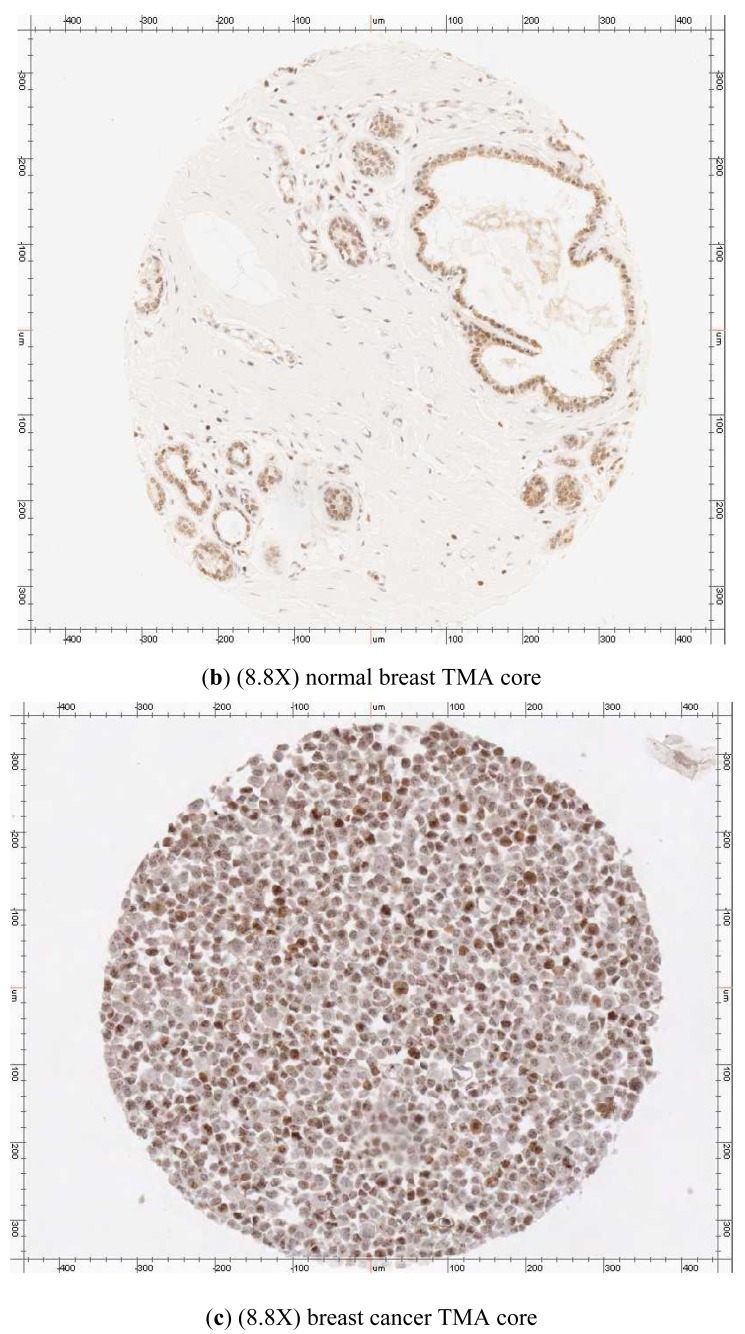

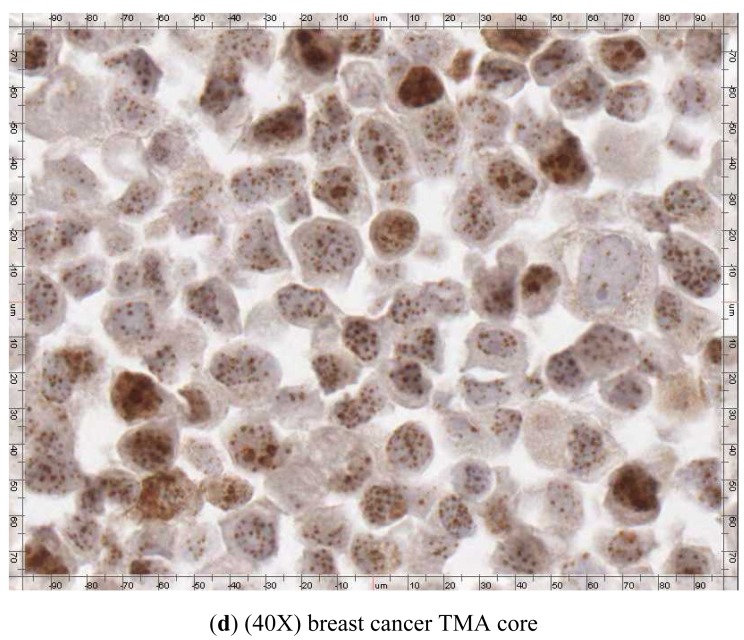
CENPA immunostaining (1:200 dilution). (**a**) Snapshot of the tissue microarray. The normal breast tissues with 20 cases (3 cores per case) are labeled in the first quadrant on the top left corner. The other 3 quadrants contain cancer samples (n = 63). (**b**) Low power (8.8X) view of normal breast tissue TMA core stained with CENPA. (**c**) Low power (8.8X) view of breast cancer TMA core stained with CENPA. (**d**) High power **(**40X) view of breast cancer TMA core stained with antibody to CENPA. The staining pattern ranged from speckled dots to pan-nuclear homogenous brown staining of the nuclei.

IHC staining for CENPA was evaluated in 20 normal breast tissue samples and 63 BCa FFPE tissue samples obtained from the Kingston General Hospital pathology department. The cases with lost cores, fewer than 5% of the area occupied by cells in the core, and ductal-carcinoma-*in-situ* were excluded from the analysis. Since three cores on the TMA represented each case, we looked at the average values per case. While we recognized that all nuclei express CENPA, the intensity of staining after optimization was very weak in some nuclei, leading to our call of no staining at 40X magnification. The samples that did stain visibly with CENPA antibody showed either discrete dots or homogenous pan-nuclear brown stain in the nuclei. The speckled pattern is typical for centromere-kinetochore proteins, while the pan-nuclear stain appeared to represent cases of high expression where the discrete speckling pattern was lost. The stain was also seen in the nuclei of the stromal cells which served as an internal positive (normal) control in all the cores studied. We scored each TMA core for percentage of visibly stained nuclei at 40X magnification. The percentage of visibly stained nuclei in BCa samples was higher compared to those in the benign breast samples (Figure 1). We categorized the average (of three cores per case) percent CENPA staining nuclei in normal and BCa samples by quartiles, as follows: 0–25%, 26–50%, 51–75%, >75%. It was found that the percentage of CENPA staining nuclei was significantly higher in BCa compared to normal breast tissue (p = 0.02) and these results are summarized in Table 2.

### 2.3. Survival Analysis

**Figure 2.**
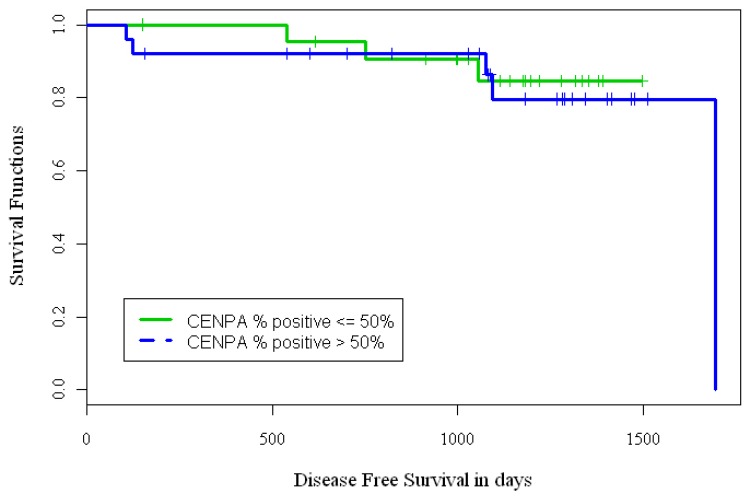
KM curves for estimating DFS in CENPA over-expressing tumors. Breast cancer patients with CENPA over-expression (>50% stained nuclei) had decreased survival (p = 0.74).

We defined three endpoints for evaluating the implications of CENPA IHC over-expression in BCa samples: overall-survival (OS), time-to-relapse (TTR) and disease free survival (DFS). However, due to the small sample size and small number of events for some endpoints, OS and TTR were not analyzed. Out of 63 BCa samples, n = 25(40%) had 0–25% nuclei stained, n = 9(14%) had 26–50% nuclei stained, n = 16(25%) had 51–75% nuclei stained, and n = 13(21%) had >75% nuclei stained with CENPA antibody. We dichotomized the CENPA IHC staining data from the BCa samples into two groups. Group 1 (low expression) included cases with 0–50% stained nuclei and group 2 (over-expression) included cases with ≥50% stained nuclei. Kaplan-Meier (KM) curves (Figure 2) were used to estimate the distributions of time to event outcomes DFS, and log-rank test was used to test difference between the low expresser (n = 34) and high expresser (n = 29) groups. For the 34 patient samples that had <50% nuclei stained with CENPA antibody, the 3-year DFS rate was approximately 85%; while for the 29 patient samples that had >50% nuclei stained with CENPA antibody, the 3-year DFS rate was 79%. The p-value from the log rank test based on categorical CENPA staining failed to achieve statistical significance possibly due to small number of patient samples on the TMA and few endpoint events (p = 0.74).

### 2.4. Kendall's Tau-b Correlation with Other Biomarkers

Using the same test groups as in the KM analysis, we evaluated the correlation coefficient between CENPA expression and ER, PR and HER2 using Kendall Tau-b. We did not find any significant correlation between expression of CENPA and these three biomarkers.

### 2.5. Univariate Analysis

In the univariate analysis, it was found that disease stage (p = 0.04) and RT (p = 0.0008) showed association with DFS. As expected, cases at later disease stage were associated with shorter DFS (HR: 5.3, 95% C.I. 1.077–26.421), while RT significantly prolonged DFS (HR: 0.05, 95% C.I. 0.008–0.285). CENPA expression trended towards association with DFS (HR: 3.4), but failed to reach statistical significance (p = 0.26). These results are summarized in Table 3.

### 2.6. Multivariate Analysis

Multivariate analysis using the Cox regression model was used to investigate the association between CENPA expression and DFS while adjusting potential confounders. We included tumor stage, ER, PR, HER2 and RT as independent predictors of DFS. The results are summarized in Table 4. Given the limited number of events and outcome information available at hand, when we included average % nuclei stained with CENPA, we observed weak evidence that higher CENPA expression was associated with worse outcome [Hazard ratio: 60.07, (low expressers vs. over expressers), p = 0.06] for DFS in BCa patients with adjusted analysis; although clearly a larger cohort will need to be examined.

### 2.7. Discussion

Aneuploidy results from gain or loss of entire chromosomes leading to genetic instability that can progress from carcinoma *in situ* or pre-cancerous lesions to invasive cancer at various sites such as the colon, cervix, and esophagus [13-15].

CENPA was first identified as an antigenic entity for autoantibody in scleroderma patients, and subsequently characterized as an 18-kDa protein [16] occupying a compact domain at the inner kinetochore plate [17]. In eukaryotes, the *CENPA* gene is well conserved [18]. Its vital role in cell division is confirmed by studies which show that mutation or knockout of *CENPA* gene results in chromosome mis-segregation [19]. Studies in a mouse model has shown that knockdown of *CENPA* is lethal in early embryonic stages [20]. The protein regulates accurate chromosomal attachment to the mitotic spindle in anaphase, signaling a delay in mitotic progression if appropriate attachment does not occur.

To the best of our knowledge, this is the first published IHC study comparing the relative expression levels of CENPA protein as assessed by IHC in both benign and malignant FFPE breast samples. We confirmed that malignant breast tissues had higher semiquantitative protein expression compared to benign tissue and that increased expression of CENPA, while not significant, trended towards association with shorter DFS and adverse outcome.

There are limited reports describing CENPA protein and mRNA levels in human cancers. Increased mRNA expression levels of *CENPA* in malignant tissue compared to its benign non-neoplastic counterpart have been described in hepatocellular carcinoma (HCC) by using RT-PCR [21]. The authors also noted a statistically significant difference in CENPA and p53 expression levels between HCC and non-neoplastic liver tissue (p < 0.01) by IHC on TMAs (n = 80). There was 88.75% (71/80) concordance between CENPA and p53 expression levels, with CENPA expression positively correlated with p53 protein levels (r = 0.57, p < 0.01). It was suggested that CENPA over-expression was regulated at the level of transcription [21].

CENPA levels were also found to be highly expressed in primary human colorectal cancer samples. *CENPA* mRNA was also up-regulated in these samples, further supporting the idea that expression of CENPA is regulated at the transcriptional level. Immunostaining with anti-CENPA antibodies showed increased CENPA signals in the tumor cells [22].

In a separate study, gene expression profiling was performed in pancreatic cancer cells treated with DUSP6/MKP-3, a specific inhibitor of *MAPK1/ERK2*. DUSP6 over-expressing cells showed down-regulation of several genes regulating mitosis including *AURKB, TPX2* and *CENPA* [23], lending further support to the idea that CENPA plays a role in carcinogenesis.

CENPA as a predictive marker is not well studied. Recently two reports have examined the association between CENPA and Holliday Junction Recognition Protein *(*HJURP*)* protein for implications in BCa therapy. HJURP and CENPA interact for correctly loading new CENPA-containing nucleosomes on the centromere, thus ensuring fidelity during chromosome segregation [24,25]. Hu *et al.* studied BCa cell lines and primary BCa cohorts and found that there was a statistically significant correlation between mRNA expression levels of *HJURP* and *CENPA* [27]. *In vitro* studies in BCa cell lines showed that RT led to increased apoptosis in cells with higher *HJURP* levels compared to cells with low *HJURP* expression levels; whereas using RNA interference studies (RNAi), it was shown that knocking down *HJURP* in human BCa cells led to decreased sensitivity to RT [26]. This indirectly provides evidence that *CENPA* mRNA expression levels may be a potential predictive marker for evaluating response to RT in BCa treatment.

Several researchers have attempted to understand the mechanism by which *CENPA* over-expression leads to aneuploidy and cancer progression. Mis-localization of CENPA leading to genome instability has been suggested [27]. In colorectal cancer cells, CENPA over-expression has been shown to result in mistargeting of the protein to noncentromeric regions, thus depleting other centromere-kinetochore components and disrupting the kinetochore complex. Another possibility is that the mistargeting of CENPA to noncentromeric regions prevents normal kinetochore assembly and thus alters the conformation of chromosome [22].

CENPA over-expression in HeLa (cervical cancer) cells was increased on the euchromatic arms of chromosomes relative to the pericentric heterochromatin [28-30]. CENPA over-expression in the Retinoblastoma protein (pRb)-depleted wild type and p53 knockout colonic epithelial cell line HCT-116 was associated with aneuploidy. To further confirm the findings, post-transcriptional silencing of *CENPA* by RNAi reduced aneuploidy and micronuclei generation in the Rb-depleted cells [8].

Considering the important role of centromeres in normal cell division and cancer, various interventions aimed at inhibiting centromere function in tumor cells are underway, the goal of which is to induce a cell cycle checkpoint and halt the spread of cancer cells. Ongoing clinical trials are investigating new anti-mitotic drugs targeting non-microtubule structures, such as mitotic kinesins and Aurora or polo-like kinases [29-33]. CENPA could be a reasonable target for such therapy, as the protein is specifically phosphorylated at the N-terminal domain in prophase, with levels peaking in prometaphase. Targeting the N-terminal domain of CENPA could potentially alter the growth activity of cancer cells, as indicated by a study where an antibody generated against the N-terminal peptide of human CENPA (residues 3–17) was microinjected into cultured cells causing cell cycle arrest in interphase, impaired cell division, and cell death [34-36].

Our study found that increased CENPA staining in BCa trended towards correlation with shorter DFS but was not statistically significant. Multivariate analysis of CENPA using tumor stage, ER, PR, HER2 and RT as independent predictors of DFS did not reveal statistically significant results. The lack of prognostic significance may be due to the limited number of CENPA stained cases, the overall small size of the TMA, an unappreciated differential effect in one or more of the underpowered intrinsic subtype groups, or heterogeneity of therapeutic treatments; and warrants further validation on a larger population–based cohort with expected proportions of current molecularly defined BCa subtypes [12]. It should also be pointed out that in prognostic signatures that currently include CENPA transcript measurements, this gene does not provide predictive power on its own, but requires the added measurement of additional genes. Therefore, while the small size of this cohort may have contributed to the observed weak correlations, it is likely that additional protein biomarkers selected from the same gene set might improve the prognostic strength.

We believe this small study, suggesting a correlation between measurable CENPA levels and disease free survival in invasive BCa, adds to our current understanding of this protein as an important component of cancer cell survival and growth. Thus, CENPA merits further investigation with respect to its biologic function and possible prognostic and/or predictive significance in breast cancer.

## 3. Experimental Section

### 3.1. Patient Cohort

With Research Ethics Board approval, breast tumor specimens were collected from 63 consenting female patients who received treatment for BCa at the Cancer Centre of Southeastern Ontario at Kingston General Hospital between 2005 and 2007. Clinico-pathological information for each case was retrospectively obtained from the medical file and entered into an anonymized database. Archival normal tissue from a cohort of twenty consenting individuals undergoing reduction mammoplasty specimens was included to provide non-malignant controls.

### 3.2. TMA Construction

We constructed a triplicate core BCa TMA in the Queen's Laboratory for Molecular Pathology. All the paraffin sections were first stained with H&E and reviewed by a pathologist. Representative tumor areas were circled and matched with the donor blocks. 0.6-mm cores were punched out from the donor blocks and embedded 1 mm apart in recipient block using Tissue Microarrayer (Beecher instruments, Silver Springs, MD).

### 3.3. Immunohistochemistry

4 μm thick TMA slides were stained with monoclonal antibody to CENPA (catalog # D115-3, clone 3–19, mouse IgG1) obtained from MBL (Medical and Biological Laboratories co., Ltd., Japan). MBL has confirmed antibody reactivity against human CENPA (17 kDa) in western blotting, immunohistochemistry and immunocytochemistry applications. A FFPE pellet of BCa cell line MDAMB-231 was used as a reference to demonstrate nuclear specificity of the antibody staining. The staining protocol was considered optimized after we observed similar nuclear staining patterns between cell line and breast tumor tissues (data not shown). Next, we evaluated normal tissue and invasive BCa whole sections for CENPA staining patterns. Technical reproducibility was tested by comparing replicate staining of two serial sections from the same TMA of a small cohort of eight tumors and four reduction mammoplasty specimens. Tumor heterogeneity was assessed by comparing stained sections from each of two test TMAs (3 + 3 cores per tumor block) from the 63-tumor cohort. The two TMAs represented different areas of the same tumor, thus allowing us to assess tumor heterogeneity. We looked at stromal cells as an internal control so as to not misclassify cases as false negative. We repeated this approach until there was 100% concordance of stained tumor cores between two serial sections on whole slides and TMA slides. Antigen retrieval was done with citrate buffer (ph 6.5) and manually stained overnight at 1:200 dilutions. The slides were also stained for ER (catalogue # 790–4324), PR (catalogue # 790–2223) and HER 2 (catalogue # 790–2991) on Ventana Benchmark automated staining system (Ventana Medical Systems, Tucson, AZ, USA) using the Ventana antibody kit (prediluted by supplier—Ventana). The detection kit used was DAB Map kit [catalogue # 760–124 (RUO)].

### 3.4. Image Acquisition

The stained slides were scanned on the Aperio ScanScope CS scanner (Aperio, Vista, CA) at 40X magnification. The slides were then analyzed with Spectrum™ digital pathology information management software and remotely viewed using freely downloadable ImageScope™ viewing software.

### 3.5. Scoring

Two pathologists, blinded to the clinical outcome, independently scored the slides manually. A senior pathologist reviewed discrepant cases. The fractions of ER and PR positive tumor nuclei were scored as 0 (<1%), 1 (1–25%), 2 (25–75%), and 3 (>75%). HER2 membranous staining was scored using Hercept test^®^ (Dako Corporation, Carpinteria, CA) scoring system as “0” if no staining was observed, or membrane staining was observed in <10% of the tumor cells; “1+” if incomplete membrane staining was observed in >10% tumor cells; “2+” if weak to moderate complete membrane staining was observed in >10% of tumor cells; “3+” if a strong complete membrane staining was observed in >10% of tumor cells. HER 2 scoring was done solely using the IHC criteria and was not confirmed with the fluorescent *in-situ* hybridization analysis. IHC staining with CENPA antibody was scored on the basis of percentage of visibly stained nuclei. The staining pattern of nuclei ranged from speckled dot-like areas to pan-nuclear brown staining at 1:200 dilutions. Whereas the normal breast cells and the low grade cancer cells showed more speckled pattern, the higher grade tumors showed more uniform homogenous pan-nuclear staining pattern (Figure 1). We categorized the average percent CENPA staining nuclei in normal and BCa tissue as follows: 0–25%, 26–50%, 51–75%, >75%. We then dichotomized with the data from the BCa samples into two groups. Group 1 (low expression) included cases with 0–50% stained nuclei and group 2 (over-expression) included cases with ≥50% stained nuclei. The staining of stromal elements inside the cores was used as a normal internal control. As triplicate cores were taken from each tumor specimen, tumor core scores were averaged to determine an overall score for each patient sample.

### 3.6. Statistical Analysis

Exploratory analyses were performed to characterize relationships between percentage of stained nuclei, with clinical factors and DFS. Kendall's tau-b correlation was used to investigate the relationships between CENPA expression levels and ER, PR and HER2. KM curves were used to estimate the distributions of time to event outcomes, and log-rank test was used to test difference between groups. Since the dataset has few deaths, we chose DFS as the primary outcome of interest. DFS was defined as the time from the date of first BCa surgery to the date of the first event of disease recurrence or death without disease recurrence, or censored at last date of disease evaluation. Cox proportional hazards models were used to evaluate the association of CENPA expression levels and DFS while adjusting for potential confounding factors.

## 4. Conclusions

This is the first study to provide a comprehensive look at the IHC expression levels of one of the key cell-cycle genes, CENPA, in FFPE breast tissue samples and its correlation with clinical outcomes. Over-expression of CENPA by IHC analysis in BCa samples trended towards an association with adverse outcome and shorter DFS. This explorative study supports further evaluation of CENPA as a potentially important protein in BCa, as both a single marker and in combination with other additional biomarkers. Some of the shortcomings of this exploratory analysis include the small sample size, lack of homogeneity of treatments, and lack of pre-specified cut-points for prospective validation. This study echoes similar findings by other groups in breast, colon and hepatocellular cancer models in predicting prognosis and response to cancer therapy. Several clinical trials with anti-mitotic drugs aimed at impairing the cell division of cancer cells are underway. Besides its possible utility as a prognostic marker in BCa, we suggest that CENPA could potentially be explored as a new therapeutic target.

## Figures and Tables

**Table 1.** Clinico-pathologic characteristics of patients included in the study.

**Parameter**	**Categories**	**Number (%))**

Age	<30	1 (2.1)
(Median: 45)	30–40	11 (22.9)
(Range: 29–49)	41–49	36 (75)

Tumor Stage	Stage 1	26(54.2)
	Stage 2	16 (33.3)
	Stage 3	1 (2.1)
	Stage 4	1 (2.1)
	Unknown	4 (8.3)

Tumor Grade	Grade 1	8 (12.7)
	Grade II	23 (36.5)
	Grade III	32 (50.8)

Lymphovascular invasion	Negative	42 (64.3)
	Positive	15 (35.7)

Number of positive nodes	0	21 (60)
	1–3	11 (31.4)
	4–10	1 (2.9)
	>10	2 (5.7)

ER Status	Negative	14 (29.2)
	Positive	34 (70.8)

PR Status	Negative	12 (25)
	Positive	36 (75)

HER2 Status	Negative	36 (75)
	Positive	9 (18.8)
	Missing value	3 (6.2)

Hormone therapy	No	14 (29.2)
	Yes	30 (62.5)
	Unknown	4 (8.3)

Radiation therapy	No	7 (14.6)
	Yes	40 (83.3)
	Unknown	1 (2.1)

Chemotherapy	No	5 (10.4)
	Yes	42 (87.5)
	Unknown	1 (2.1)

**Table 2.** CENPA staining by tissue type.

**Tissue Type**	**#Patients**	**0–25% stained nuclei**	**26–50% stained nuclei**	**51–75% stained nuclei**	**>75% stained nuclei**	**P-Value 1**
Normal	20	13 (65%)	5 (25%)	2 (10%)	0 (0%)	
Breast cancer	63	25 (40%)	9 (14%)	16 (25%)	13 (21%)	0.02

1 P-value by exact Fisher test. Average percentage of nuclei stained with antibody to CENPA were categorized as follows: 0-25%, 26-50%, 51-75%, >75%. Percentage of nuclei stained with antibody to CENPA was significantly higher in breast cancer compared to normal breast tissue (p = 0.02).

**Table 3.** Univariate analysis showing hazard ratios (HR), Confidence intervals (C.I.) and P values in the breast cancer patient cohort.

**Parameter**	**Parameter Estimate**	**Hazard Ratio**	**95% C.I. for HR**	**P-value**
Age	0.01077	1.011	0.878–1.164	0.8813
Stage	1.67415	5.334	1.077–26.421	0.0403
CENPA	1.23581	3.441	0.315–21.892	0.2564
ER	−0.04795	0.953	0.185–4.924	0.9544
PR	−0.24916	0.779	0.151–4.023	0.7660
HER2	0.47485	1.608	0.311–8.303	0.5708
Hormone Therapy	0.95719	2.604	0.313–21.637	0.3756
Radiation Therapy	−3.02405	0.049	0.008–0.285	0.0008
Chemotherapy	15.15319	3810184	0.000–∞	0.9950

**Table 4.** Cox proportional model to predict disease free survival. Multivariate analysis showing hazard ratios (HR), Confidence intervals (C.I.) and P values in the breast cancer patient cohort.

**Parameter**	**Parameter Estimate**	**Hazard Ratio**	**95% C.I. for HR**	**P-value**
CENPA	4.09553	60.071	0.303–130.022	0.0601
Stage	4.69694	109.611	2.583–990.042	0.0087
ER	4.78711	119.955	0.283–262.032	0.0639
PR	−5.00491	0.007	0.001–1.037	0.0315
HER2	4.13198	62.301	0.642–522.440	0.0450
Radiation Therapy	−4.41129	0.012	0.001–0.189	0.0024
